# Excision of an Unexpected Retroperitoneal Mass at Laparoscopy

**DOI:** 10.7759/cureus.87839

**Published:** 2025-07-13

**Authors:** Tejal Date, Abhijit Aich, Haissam Moukarram

**Affiliations:** 1 Obstetrics and Gynaecology, North Cumbria Integrated Care, National Health Service (NHS) Trust, Whitehaven, GBR

**Keywords:** adnexal masses, ovarian cyst, ovarian dermoid cyst, pelvic masses, solitary neurofibroma

## Abstract

Neurofibroma is a benign soft tissue tumour of neural origin. Neurofibromatosis or Von Recklinghausen disease is an autosomal-dominant condition. Pelvic occurrence of a solitary neurofibroma is uncommon.

A 54-year-old woman presented with a history of chronic back pain with occasional dyspareunia for 12 months. She had a hysterectomy and right salpingo-oophorectomy for endometriosis many years back. Clinical examination revealed fullness and tenderness at left vaginal vault. Pelvic ultrasonography revealed a 55x49x55mm mass suggestive of dermoid cyst. Magnetic resonance imaging findings were consistent with dermoid cyst. Intraoperatively, a retroperitoneal mass in the left pelvic sidewall was removed along with left fallopian tube and ovary. Histopathology revealed neurofibroma. There was no recurrence on follow-up.

Solitary, de novo, retroperitoneal pelvic neurofibroma is very rare. A lack of familiarity with the anatomy and pathogenesis of retroperitoneal masses often leads to a diagnostic conundrum due to overlapping imaging findings. A possibility of neurofibroma or schwannoma should be kept in mind while evaluating pelvic and/or adnexal masses.

## Introduction

Adnexal masses are commonly encountered by obstetricians and gynaecologists and can be found in women of all ages. These masses could be of gynaecological or non-gynaecological origin and present diagnostic and management dilemmas [1]. Adnexal masses of gynaecological origin could be ovarian benign or malignant lesions, tubal, para-tubal or tubo-ovarian masses like ectopic pregnancy, hydrosalpinx, para-tubal cysts, tubo-ovarian abscesses and uterine abnormalities such as fibroids and Mullerian anomalies [1]. Often, these masses are detected incidentally at the time of imaging or physical examination. Patients with these masses may present with pain in abdomen intermittent, acute or chronic, dysfunctional uterine bleeding and postmenopausal bleeding. Though most of the masses are benign, the main aim of diagnostic evaluation is to rule out malignancies [1]. Management options are guided by diagnostic findings, detailed history and willingness for fertility preservation.

Pelvic retroperitoneal masses are relatively rare. There is also a lack of familiarity with the anatomy and pathogenesis of these masses, which often lead to a diagnostic conundrum due to overlapping imaging findings [2]. The clinical presentations of retroperitoneal masses, depending on the location and surrounding structures, are non-specific. The common presentation of retroperitoneal tumours is poorly localised pain, which may also be present in lower extremities or in the genitalia, accompanied by tingling, numbness and/or urinary symptoms. At times, large retroperitoneal masses can cause pressure symptoms on visceral and genito-anal organs [3].

Neurofibroma is a benign tumour of neural origin. It is commonly seen as a partial manifestation of neurofibromatosis type 1 (NF-1) [4]. Neurofibroma can be cutaneous, spinal or plexiform, with plexiform type tending to turn malignant [5]. Neurofibromatosis or Von Recklinghausen disease is an autosomal-dominant inheritable disorder. The gene encodes neurofibromin, which is expressed widely in a variety of tissues and acts as a tumour suppressor gene. The deletion or mutation of this gene results in the proliferation of multiple neurofibromas and other tumours. Retroperitoneal pelvic neurofibromas are rare [5]. Neurofibromatosis type 1 is associated with multiple neurofibromas, café au lait spots and Lisch nodules. Multiple organ systems involved are skin, vascular, endocrine, eyes, gastrointestinal, central and peripheral nervous systems. Most symptoms appear at the age of 10 years. Some cutaneous signs like freckles may appear from birth to five years [6].

As gynaecologists, our encounter with retroperitoneal masses is relatively rare and is often misdiagnosed as uterine or ovarian masses. We report a case of retroperitoneal neurofibroma mimicking a dermoid cyst.

## Case presentation

A 54-year-old white woman presented with dyspareunia, chronic pelvic discomfort, backache and intermittent constipation. She had no pressure symptoms, systemic presentations or neurological deficit. The patient had a total abdominal hysterectomy with right-sided salpingo-oophorectomy for endometriosis. She had no family history of cancer. 

On examination, the patient had normal body mass index and no dermal stigmata of neurofibromatosis. Abdominal and speculum examinations were unremarkable. On bi-manual examination a firm, regular, tender mass was noted on the left side of vaginal vault. Per rectal examination was normal.

**Figure 1:**
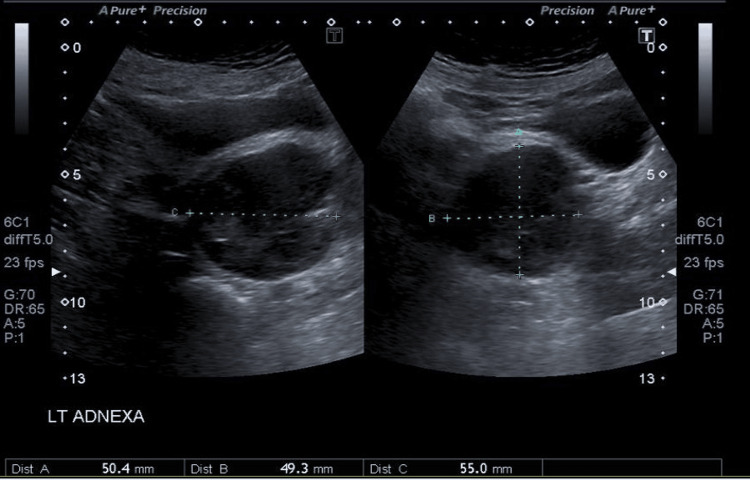
Ultrasound image demonstrating 50x49x55 mm cystic mass adjacent to left ovary. Hyper echoic areas in the cyst are consistent with features of dermoid cyst.

**Figure 2:**
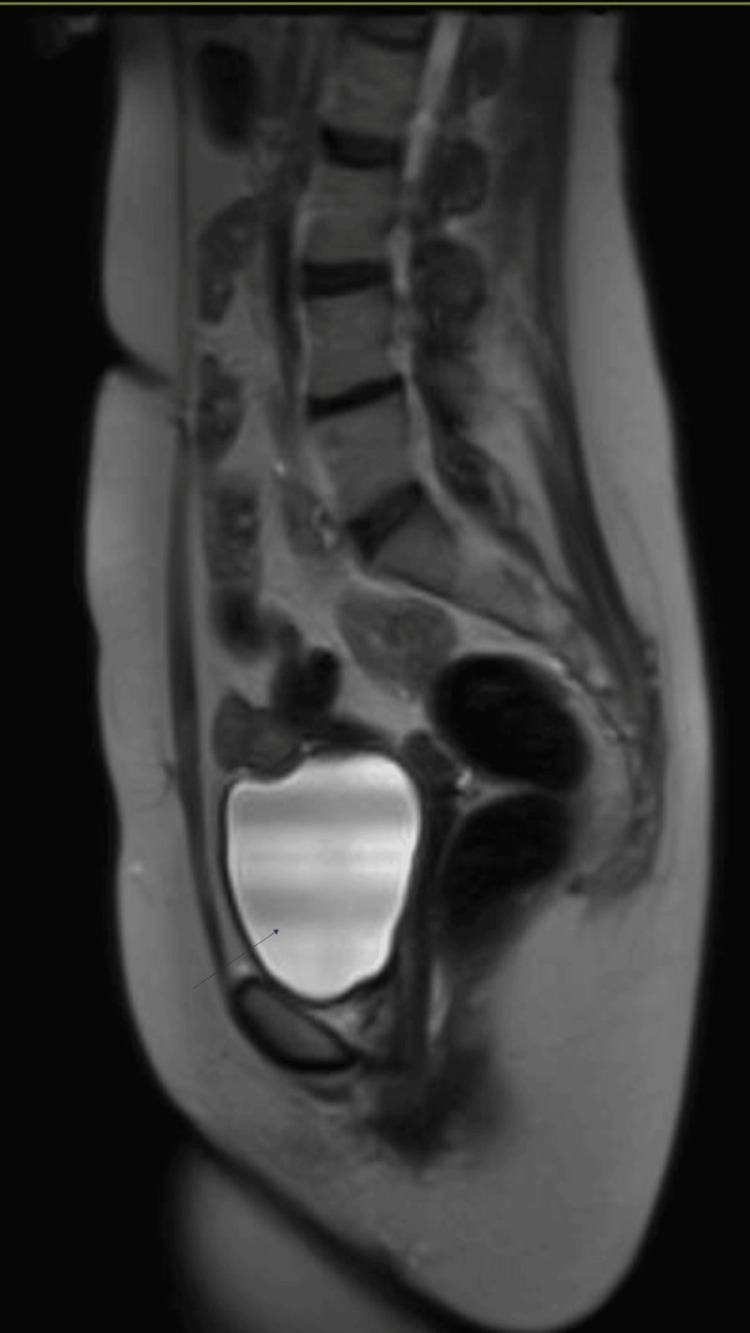
MRI sagittal plane: Heterogenous cystic mass in left adnexa, adjacent to vaginal vault consistent with dermoid cyst as evidenced by small area of lipid content within the lesion. Hyperintense area seen on T2-weighted image.

All haematogical and biochemical tests and tumour markers were normal. Pelvic ultrasonography (Figure 1) and magnetic resonance imaging (Figure 2) were suggestive of a dermoid cyst measuring 55x49x55mm. 

A provisional diagnosis of benign ovarian tumour with a risk of malignancy index <200 was made. After ureteric stenting, laparoscopic left salpingo-oophorectomy was done. Intraoperatively, a well-encapsulated retroperitoneal mass was confirmed. A provisional diagnosis of lipoma was made. The mass was removed with left ovary and tube.

Histopathological findings were consistent with that of neurofibroma. Immunohistochemistry staining was positive for vimentin and S100. There was no evidence of malignancy. The findings were confirmed by the consultant pathologist at a tertiary centre. A complete excision of neurofibroma was achieved.

**Figure 3:**
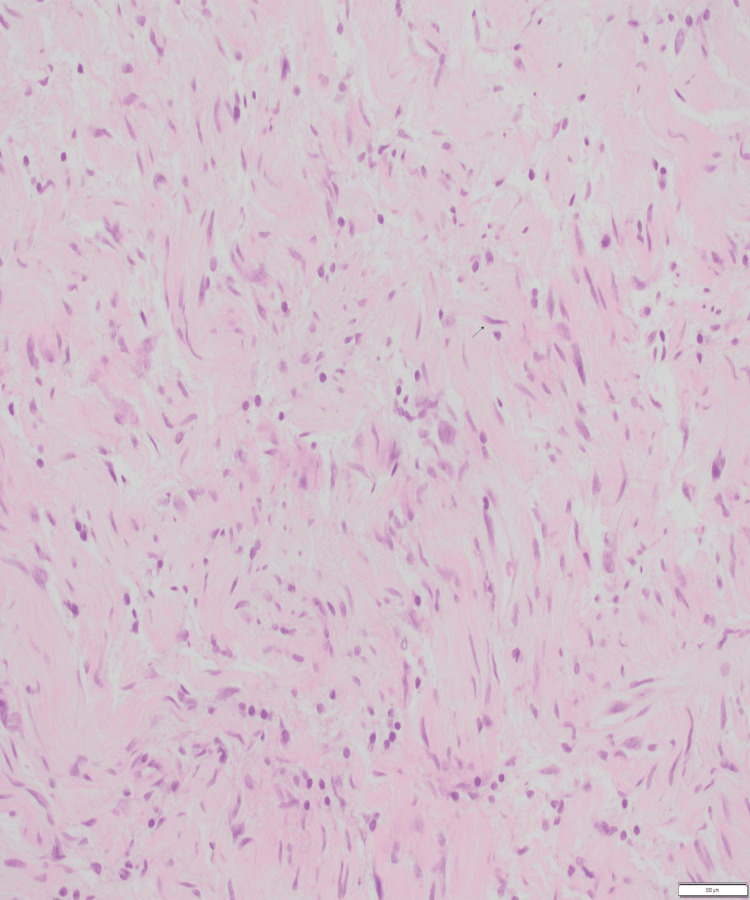
Histology section shows spindle cell proliferation

**Figure 4:**
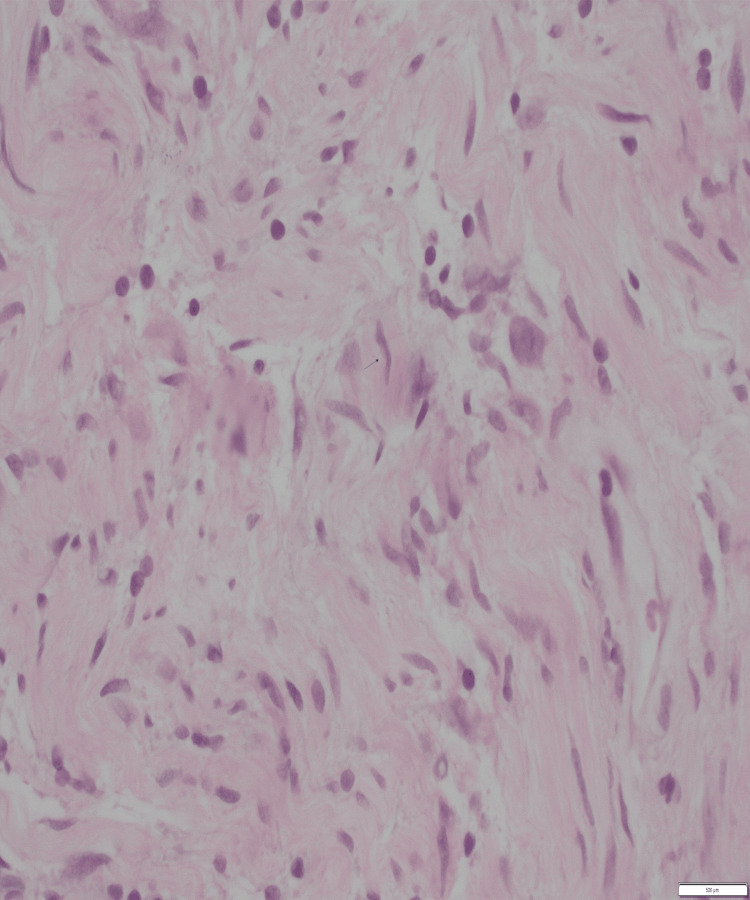
On higher power (400x), the cells have distinctive cytoplasm and elongated wavy nuclei

**Figure 5:**
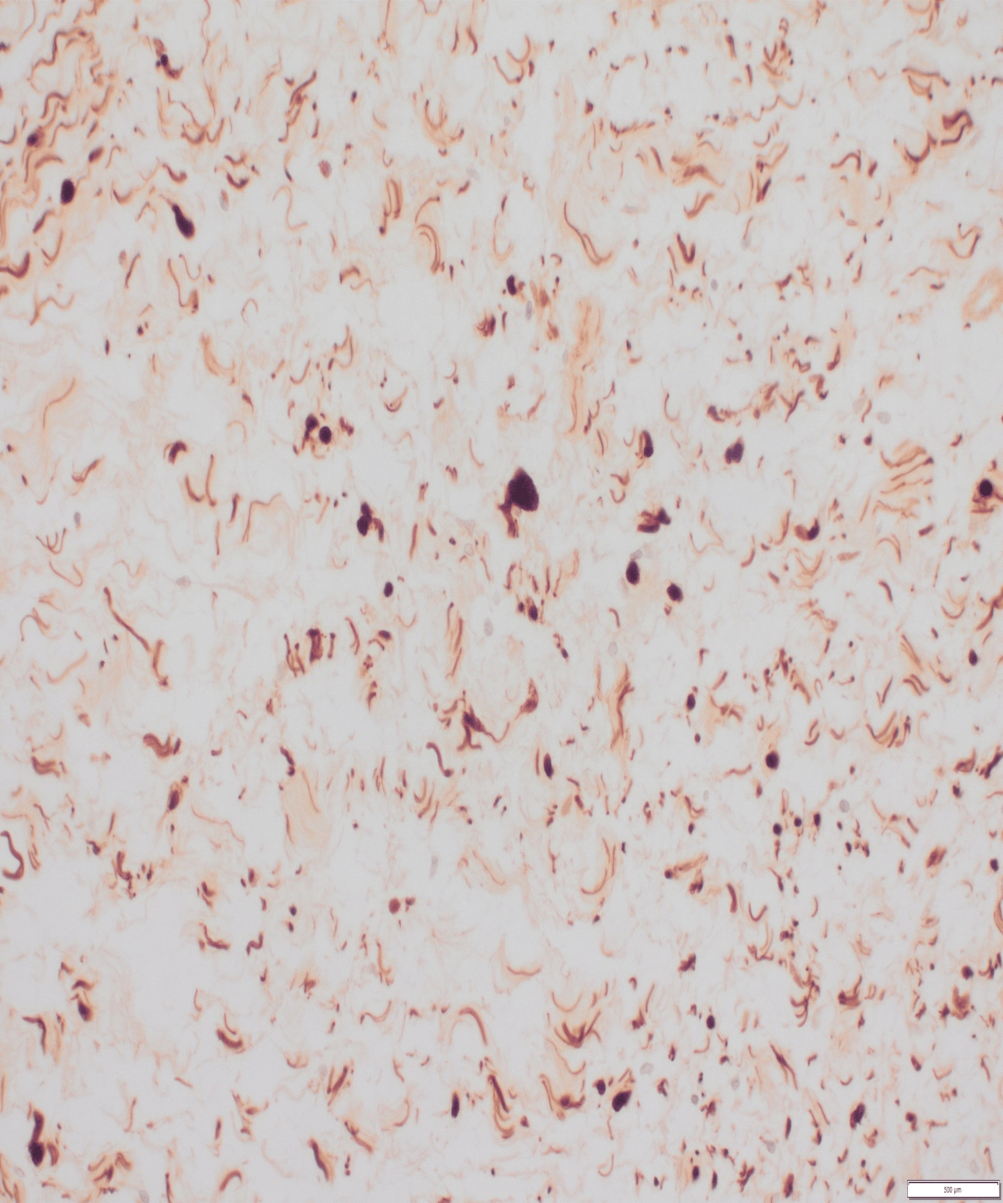
On immunohistochemistry, the spindle cells are positive for S100

Figure 3 shows spindle cell proliferation consistent with neurofibroma. Figure 4 on higher power shows cells with indistinct cytoplasm and elongated nuclei and Figure 5 shows spindle cells positive for S100 on immunohistochemistry.

At one-year follow-up, the patient was symptom-free and there was no evidence of disease recurrence.

## Discussion

Adnexal masses could be of gynaecological or non-gynaecological origin. Most of the masses are benign. The aim of diagnostic evaluation is to rule out malignancies [1]. The prevalence of ovarian cysts in postmenopausal patients is 5%-17% [7]. The lifetime risk for surgery for suspected ovarian neoplasm is 5%-10% [8]. Dermoid cyst accounts for 10%-20% of all ovarian tumours and is the most common benign neoplasm [9].

There is a lack of familiarity with the anatomy and pathogenesis of retroperitoneal masses, which lead to a diagnostic conundrum [10]. The clinical presentations of retroperitoneal masses are non-specific. Large retroperitoneal masses can cause pressure symptoms [2]. Our patient did not report any pressure symptoms.

Neurofibroma can be cutaneous, spinal or plexiform, with the latter tending to turn malignant [5]. The diagnosis of NF-1 depends on the clinical presentation of signs and symptoms. Our patient did not have any dermal stigma, no optic glioma or osseous lesions and no family history of NF-1.

Topsakal et al. presented a case of solitary pre-sacral neurofibroma in a patient initially diagnosed as large right ovarian mass and presented with bilateral chronic sciatica [3]. Our patient did not have any neurological deficit. Chao et al. reported a case of solitary neurofibroma involving the obturator nerve in a woman with right-sided pelvic mass mimicking a complex ovarian tumour [2].

Benign peripheral nerve sheath tumours called Schwannomas can also be found in the pelvis. Schwannomas can be easily shelled while preserving the nerve. However, in most neurofibromas, the nerve is incorporated within the mass, which is resected and warrants subsequent nerve graft to restore the function [4]. Ching-Hui Chen et al. reported a case of pelvic mass initially diagnosed as a uterine fibroid but later proven to be a retroperitoneal cellular schwannoma on histology [11]. Vulval and myometrial neurofibromatosis [12] has been reported in the past but it has rarely been reported in context of vagina, ovaries or cervix.

## Conclusions

Retroperitoneal pelvic masses are rarely encountered in gynaecological practice. A lack of familiarity with the anatomy and pathogenesis of retroperitoneal masses often leads to a diagnostic conundrum. Pelvic neurofibroma is uncommon and can be misdiagnosed as uterine, ovarian or tubal pathology. A possibility of neurofibroma or Schwannoma should be kept in mind while evaluating pelvic and/or adnexal masses. This case report shows that solitary pelvic neurofibroma can arise de novo without any family history, genetic disorder, obvious dermal stigmas or organ involvement. Imaging permits better characterisation, localisation of tumours and therefore is an essential tool in making a diagnosis or planning treatment.
